# Practical use, effects and complications of prehospital treatment of acute cardiogenic pulmonary edema using the Boussignac CPAP system

**DOI:** 10.1186/1865-1380-6-8

**Published:** 2013-04-08

**Authors:** Eva Eiske Spijker, Maarten de Bont, Matthijs Bax, Maro Sandel

**Affiliations:** 1 Emergency Department, Haga Hospital, Leiden University, The Hague, The Netherlands; 2 Present: Leiden University Medical Center, P.O. Box 9600, 2300 RC Leiden, The Netherlands; 3 Emergency Department, Erasmus MC, P.O. Box 2040, 3000 CA Rotterdam, The Netherlands; 4 Cardiology Department, Haga Hospital The Hague, 275, 2545 CH The Hague, The Netherlands; 5 Emergency Department, Haga Hospital The Hague, Leyweg 275, 2545 CH, The Hague, The Netherlands

**Keywords:** Non-invasive ventilation, Continuous positive airway pressure (CPAP) ventilation, Boussignac mask, Acute heart failure, Acute cardiogenic pulmonary edema, Prehospital emergency care

## Abstract

**Background:**

Early use of continuous positive airway pressure (CPAP) has been shown to be beneficial within the setting of acute cardiogenic pulmonary edema (ACPE). The Boussignac CPAP system (BCPAP) was therefore introduced into the protocols of emergency medical services (EMS) in a large urban region. This study evaluates the implementation, practical use and complications of this prehospital treatment.

**Methods:**

This was a retrospective case series study. The study was carried out in a period shortly after the implementation of the BCPAP system on all EMS ambulances in the The Hague region. According to protocol, diagnosis of ACPE in the prehospital setting was left to the discretion of the EMS paramedics and the facial mask was applied immediately after the diagnosis had been made. Patients were selected through hospital registration and diagnostic criteria for ACPE. Only those patients showing evident clinical signs of ACPE were included. Patient characteristics, physiologic variables, clinical outcomes and complications were collected from EMS transport reports and hospital records.

**Results:**

Between 1 June 2008 and 30 April 2009 a total of 180 patients were admitted for ACPE. Of these, 76 (42%) had evident clinical signs of ACPE upon presentation and were included. Three patients were transferred and in 14 cases data were missing. Out of the remaining 59 patients, 16 (27%) received BCPAP. In 43 (73%) cases the mask was not applied. For 7 out of 43 cases that were eligible for BCPAP treatment but did not receive the facial mask, an explanation was found in the EMS transport record. No complications were recorded pertaining to using the BCPAP system.

**Conclusions:**

A significant portion of patients with clinical signs of acute cardiogenic pulmonary edema in the prehospital setting is not treated according to protocol using BCPAP. Based on the small group of patients that actually received BCPAP treatment, the facial mask seems feasible and effective for the treatment of acute cardiogenic pulmonary edema in the prehospital setting.

## Background

Early use of continuous positive airway pressure (CPAP) has proven to be beneficial in preventing endotracheal intubation and reducing intensive care unit (ICU) and coronary care unit (CCU) length of stay within the setting of acute cardiogenic pulmonary edema (ACPE) [1]. The Boussignac CPAP (BCPAP) facial mask system is a compact and flexible CPAP system that can be used by emergency medical service (EMS) paramedics in the prehospital setting [2-4]. A prospective study with BCPAP performed at a coronary care unit in The Netherlands reported a 14 per cent decrease in the number of endotracheal intubations, 25 per cent decrease in ICU admissions, 50 per cent decrease in ICU length of stay and 50 per cent decrease in median hospital length of stay [5]. Several studies also showed that the early use of CPAP in the prehospital setting was associated with a better outcome [5]. Regional implementation of the BCPAP system into the standard ACPE protocols of the emergency medical services took place recently. The aim of this study was to evaluate the use, effects and complications of the BCPAP protocol following implementation into the regional EMS of The Hague, The Netherlands.

## Methods

This was a retrospective observational case series study.

### The Boussignac CPAP system

**Figure 1:**
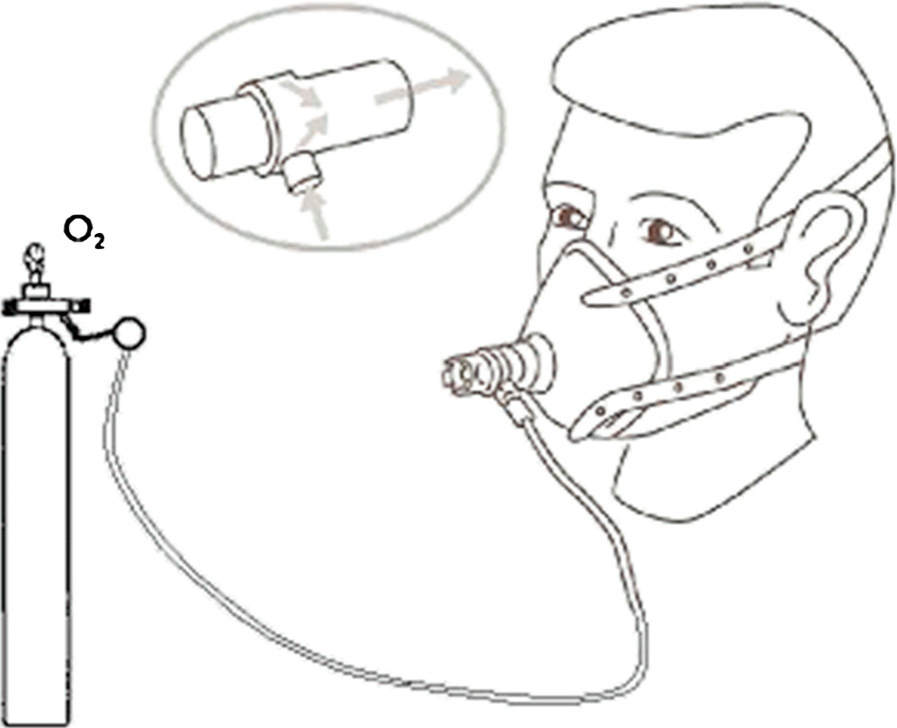
Application of the Boussignac CPAP mask.

**Figure 2:**
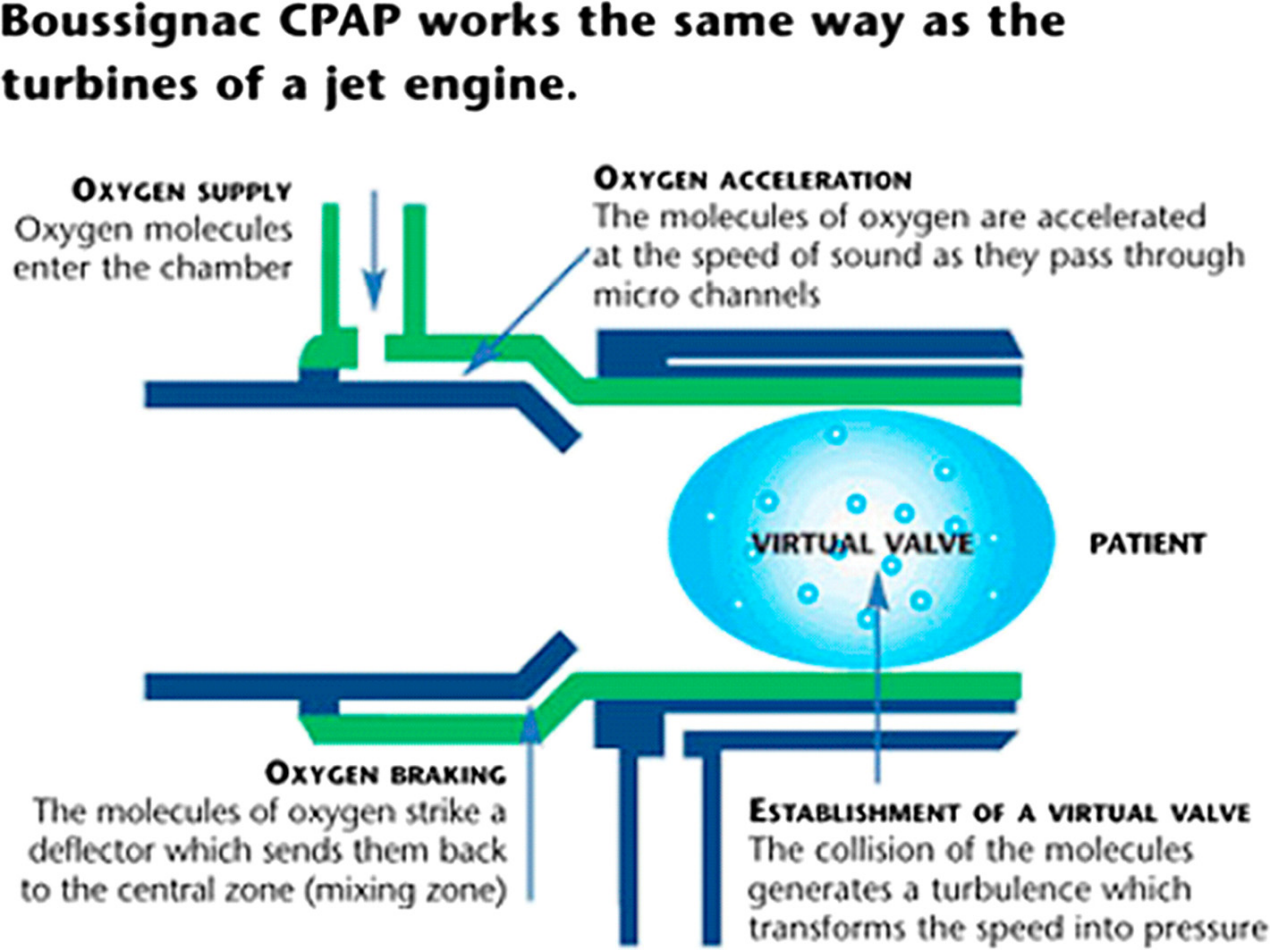
Direction of the oxygen flow in the Boussignac CPAP mask.

The Boussignac CPAP system is a simple, safe, cheap and lightweight (10 g) plastic cylinder that is directly connected to a disposable face mask (Figure 1[6]) and has been shown to be effective for ACPE in the emergency department and in prehospital care [2-4]. The Boussignac system provides continuous positive airway pressure by injecting oxygen at high speed into a cylinder through curved side channels. The resulting turbulence in combination with air friction drives air into the cylinder, causing it to flow along the insides of the cylinder (Figure 2[7]). The system is lightweight because there is no need for ventilators or machines with main connection or heavy tubing. An oxygen flow of 15 l/min was used to generate a pressure of 5 cm H_2_O.

### Setting

**Figure 3:**
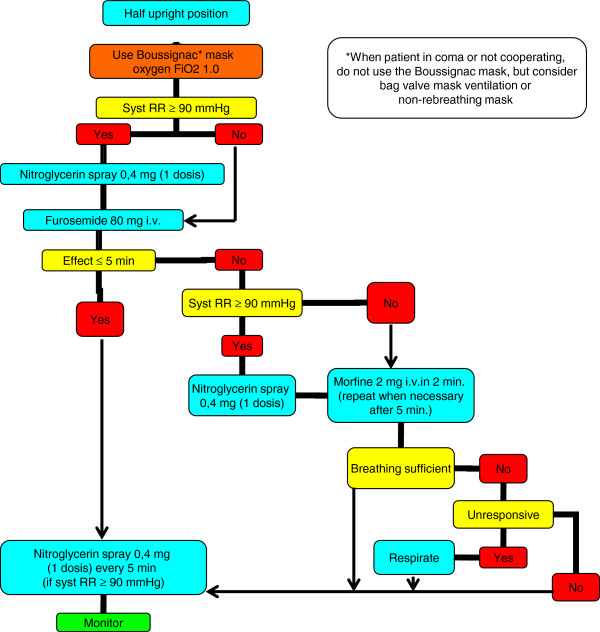
National EMS protocol for acute cardiogenic pulmonary edema treatment.

This study was performed at the Haga Hospital and regional EMS organisations in The Hague, The Netherlands. The emergency department (ED) has an annual visit of 45,000 patients, cooperates with two regional EMSs and covers a rural and industrial area with a population of ±500,000 [8]. BCPAP was officially introduced on all EMSs in June 2008. All EMS paramedics participated in an extensive practical training and two E-learnings on using the mask. Indications and contraindications for using the BCPAP were discussed. Contraindications for applying the mask were decreased consciousness, facial trauma and anatomical variations (e.g. tracheotomy, bearded men) that made application of the mask impossible. All EMS ambulances in the region were equipped with the disposable BCPAP system with different mask sizes. Application of the BCPAP mask was left to the discretion of the EMS paramedics. All patients were treated on an intention-to-treat basis and received diuretics and vasodilators according to protocol. This study was reviewed and approved by our institutional research board. The national EMS protocol for acute cardiogenic pulmonary edema is shown in Figure 3.

### Study design

We used hospital registration to select all patients diagnosed with ACPE upon presentation at the emergency department from 1 June 2008 until 30 April 2009. We excluded those cases that did not meet all ACPE criteria, i.e. acute dyspnoea within the preceding 24 h, or acute worsening of dyspnoea and crepitations of both lungs upon auscultation. All vital parameters were retrieved from the EMS ambulance transport records. Diagnostic and therapeutic considerations from EMS personnel were also recorded.

### Outcome measures

Primary outcome parameters were the number and percentage of eligible ACPE patients that were treated using the BCPAP system within the prehospital setting. Secondary outcome parameters were complications (aspiration, mask discomfort or pain), feasibility of mask application, changes in physiologic variables, endotracheal intubation rate, ICU, CCU and hospital lengths of stay, and hospital mortality.

### Primary data analysis

Statistical analysis was performed using SPSS 16.0.01, SPSS, Chicago, IL, USA.

Since most outcome measures did not show normal distribution, we used nonparametric tests for the statistical analysis. We used the Mann–Whitney *U* test to analyse numerical data, the chi-square test or Fisher’s exact test (when a cell count was less than five) for categorical data. Significance level was set as *p* < 0.05. Power analyses were not performed for secondary measures.

## Results

### Main outcomes

**Figure 4:**
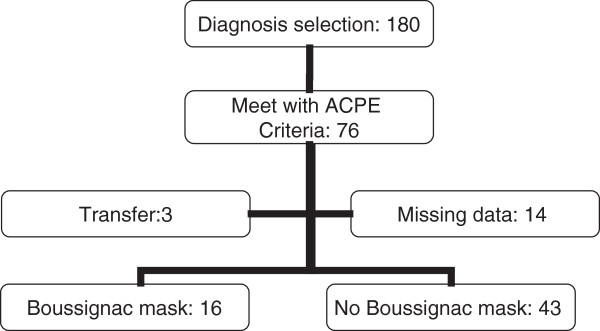
Flowchart of included patients.

Between 1 June 2008 and 30 April 2009, 180 patients were diagnosed with ACPE upon admission. Seventy-six cases met our ACPE criteria. Three patients were transferred and in 14 cases data were missing. Of the remaining 59 patients, 16 (27%) received BCPAP before arrival at the emergency department. In 43 (73%) cases, the mask was not applied (Figure 4). For 7 out of 43 cases that were eligible for BCPAP treatment but did not receive the facial mask, an explanation was found in the EMS transport record. In two cases problems were noted in the practical use, in three cases the mask was not applied because the patient was agitated, and in two cases there were patient-related contraindications, i.e. tracheotomy and a nosebleed. In the remaining 36 cases no explanation was given for not giving BCPAP in the prehospital setting. No adverse effects (e.g. facial skin injury, pneumothorax) related to the BCPAP were noted during this study.

### Explanations for failed BCPAP administration

In 7 cases BCPAP treatment was not administered and an explanation was found in the EMS transport records. Twice the explanation referred to a dysfunctional system (i.e. ‘system is not working’ and ‘system leaks at tank’). Twice the EMS personnel stated that the patient was too agitated to accept the mask. Other explanations were ‘patient did not accept mask’, ‘tracheotomy’ and ‘nosebleed’.

### Characteristics of study subjects

Patients’ ages ranged from 77 to 93 years versus 55 to 95 years in the BCPAP group versus the conventional treatment group, both with an even gender distribution. Duration of prehospital treatment ranged from 20 to 47 min in the BCPAP group and 15 to 87 min in the non-BCPAP group. This was measured as the interval between arrival of ambulance personnel at the patients’ location and arrival at the emergency department.

Eighteen patients are not included in the analysis of prehospital treatment time because the documentation of the treatment time was incomplete, i.e. time of arrival at patient’s location, time of arrival at emergency department or both were missing on the patients’ ambulance transport records (Table 1).

**Table 1:** Patients’ characteristics, precipitating factors and prehospital features

Characteristic in median ± IQR unless otherwise stated	Patients received BCPAP (*N* = 16 unless otherwise stated)	Patients did not receive BCPAP (*N* = 43 unless otherwise stated)	*p*-value
Age, years	85 ±10	84 ± 10	0.26
Male (%)	50	47	0.81
History			
History of admission for CHF, %	19	16	1.0
History of ACS, %	38	33	0.72
History of AP, %	25	14	0.44
History of CHF, %	31	37	0.67
History of COPD, %	13	14	1.0
Precipitating factors			
AMI with ST elevation, %	0	0 (*N* = 41)	-
AMI without ST elevation, %	31	19 (*N* = 41)	0.48
No AMI, %	69	77 (*N* = 41)	0.48
Prehospital features			
Pulse oximetry, %	70 ± 22 (N = 13)	88 ±11 (*N* = 32)	0.002
Heart rate, beats/min	111 ± 28 (*N* = 11)	110 ± 31 (*N* = 30)	0.25
Systolic blood pressure, mmHg	169 ± 35 (*N* = 13)	162 ± 50 (*N* = 30)	0.60
Diastolic blood pressure, mmHg	104 ± 32 (*N* = 13)	98 ± 34 (*N* = 30)	0.58
Prehospital treatment time, min	30 ± 11 (*N* = 11)	39 ± 20 (*N* = 30)	0.08

*IQR*, interquartile range; *BCPAP*, Boussignac continuous positive airway pressure; *CHF,* congestive heart failure; *ACS*, acute coronary syndrome; *AP*, angina pectoris; *COPD*, chronic obstructive pulmonary disease; *AMI*, acute myocardial infarction; *ED*, emergency department. When data are missing, this is stated in the table.

### Clinical parameters and in-hospital outcomes

In the BCPAP group the median pulse oximetry measured by the ambulance personnel on arrival at the patient’s location was 70% and had increased to 95% upon arrival at the ED. There was no major change in median heart rate after BCPAP treatment. Median systolic and diastolic blood pressure showed a decrease.

In those patients who did not receive BCPAP, the median pulse oximetry increased from 88% to 95%. The median heart rate, systolic and diastolic blood pressure decreased slightly during the conventional prehospital treatment. One patient who received BCPAP (6%) and three patients who did not receive BCPAP (7%) were intubated during admission. In the BCPAP group one patient (6%) was admitted to the ICU for a total of 53 h and 14 patients (88%) were transferred to the CCU after evaluation at the ED. Median length of stay at the CCU was 23 h. In the conventional treatment group, five patients (12%) were admitted to the ICU with a median length of stay of 92 h. Twenty-two patients (54%) were treated at the CCU after evaluation at the ED. This group had a median length of stay at the CCU of 23 h as well.

Among those patients who survived to hospital discharge, the length of stay ranged from 1.5 to 20 days, with a median of 4.6 days, and from 1.5 to 34 days, with a median of 5.1 days for the BCPAP and conventional treatment group, respectively. Two patients who received BCPAP (12.5%) and four patients who did not receive CPAP (9.3%) died during admission.

The groups were comparable with regard to demographics, precipitating factors and prehospital vital signs, except for the median pulse oximetry measured by the ambulance personnel upon arrival at the patient’s location, which was significantly lower for the BCPAP group (*p* = 0.002). Characteristics are summarised in Tables 1 and 2.

**Table 2:** Patients’ treatment and in-hospital outcomes

**Characteristic in median ± IQR unless otherwise stated**	**Patients received BCPAP (*N *= 16 unless otherwise stated)**	**Patients did not receive BCPAP (*N *= 43 unless otherwise stated)**
**Treatment prehospital**		
Furosemide, %	100	63 (*N* = 37)
GTN sublingually, %	88 (*N* = 15)	47 (*N* = 34)
Intubation, %	0	0 (*N* = 39)
**In-hospital outcomes**		
Pulse oximetry, %	95 ± 11	95 ± 7 (*N* = 38)
Heart rate, beats/min	114 ± 35	98 ± 33
Systolic blood pressure, mmHg	160 ± 40	151 ± 49 (*N* = 41)
Diastolic blood pressure, mmHg	86 ± 19	80 ± 33 (*N* = 41)
O_2_ content first ABG, %	97 ± 6	96 ± 6 (*N* = 29)
paCO_2_ in first ABG, kPa	6.2 ± 2.8	5.9 ± 2.1 (*N* = 29)
paO_2_ in first ABG, kPa	12.1 ± 20	11.3 ± 4.2 (*N* = 29)
pH in first ABG	7.3 ± 0.1	7.3 ± 0.1 (*N* = 29)
Intubation, %	6.3	7.0 (*N* = 41)
Admission to ICU, %	6.3	12 (*N* = 42)
Length of stay ICU, hours	53 (*N* = 1)	24 ± 196 (*N* = 5)
Admission CCU, %	88	54 (N = 42)
Length of stay CCU, hours	23 ± 13 (*N* = 14)	23 ±32 (*N* = 22)
Length of stay total, days	4.6 ± 3.9 (*N* = 15)	5.1 ±7.3 (*N* = 39)
In-hospital mortality,%	12.5	9.3

*IQR*, inter quartile range; *BCPAP*, Boussignac continuous positive airway pressure; *GTN*, glyceryl trinitrate; *ABG*, arterial blood gas; *CCU* = coronary care unit.

## Discussion

Our observational study showed that a significant portion of patients with evident signs of acute cardiogenic pulmonary edema within the prehospital setting did not receive BCPAP according to protocol. Only a minority of eligible patients received the BCPAP treatment from the EMS personnel. Moreover, only in 16 per cent of cases that did not receive BCPAP was a documented reason found. No complications were reported pertaining to the use of the facial mask.

The benefits of CPAP treatment for ACPE are well established in the emergency eepartment and ICU, showing significant improvement of vital parameters and reduction in endotracheal intubations, ICU/CCU admissions and mortality [1].

The Boussignac CPAP system is a compact and easy to use system, the feasibility and effectiveness of which within the prehospital setting have been shown in several studies [2-4]. Several studies reported that early treatment of ACPE using BCPAP reduced the number of endotracheal intubations and decreased ICU, CCU and hospital lengths of stay [5,9-13].

Even though this study did not focus on evaluating outcome parameters of the BCPAP, we present these parameters in Table 2. There are insufficient data in this study to report difference in outcome. It is unclear if the outcomes observed reflect efficacy of the BCPAP versus no BCPAP, as the study did not aim to establish improvement in outcome. However, future studies in our population should be designed to assess whether outcome differences exist.

The EMS organisations in The Hague region implemented the BCPAP system in their standard protocol for treatment of ACPE. This observational study evaluated the use, effects and complications of BCPAP treatment by EMS personnel.

It is well known that ACPE is difficult to diagnose. Generally, differentiation between clinical signs of COPD and ACPE is considered to be difficult. When clinical signs were not obvious upon admission, the diagnosis ACPE was often supported by ancillary tests. However, we chose to exclusively include patients on clinical grounds, without benefit of chest radiography or laboratory results, to simulate diagnostic conditions in the field. Some studies suggested that diagnosis of ACPE in the prehospital setting is too difficult for EMS paramedics. Templier et al. studied the identification of ACPE by trained emergency nurses and physicians. The results show that nurses can recognise ACPE equally well as physicians, who correctly diagnosed ACPE in 77% of cases [4].

It is unclear why a significant portion of cases did not receive BCPAP treatment according to protocol in our study. A documented reason for not complying with the protocol was only given in 16 per cent of cases. Since the study period was shortly after the introduction, it is tempting to suggest that EMS personnel were not familiar with the treatment, or used the mask for the more severely hypoxic patients, as the median pulse oximetry in the BCPAP group was significantly lower. This could mean that a more intensive training programme is warranted to increase the use of BCPAP. EMS personnel also claimed that the mask caused discomfort for the patient and inconvenience when administering glyceryl trinitrate sublingually, as the mask has a tight fit. Regarding the mask causing discomfort, EMS paramedics affirm experiencing fewer problems when they made an effort to prepare and coach the patient to leave the mask in place. In some cases application was not possible as described in the results, but no complications were noted during treatment with the Boussignac mask.

### Limitations

The present study has several limitations. Firstly, data were collected retrospectively. Through stated criteria of inclusion, based on established criteria, we ensured an accurate selection of patients [1]. Secondly, we compared patient characteristics of cases that were actually treated in conformity with the BCPAP protocol and cases that did not receive BCPAP treatment. The factors contributing to the decision to treat patients using BCPAP are largely unknown. Therefore, we cannot exclude ascertainment biases in this analysis. Thirdly, the study contains a limited number of patients.

## Conclusion

A significant portion of patients with clinical signs of acute cardiogenic pulmonary edema in the prehospital setting is not treated according to protocol using BCPAP. No complications were reported pertaining to using the BCPAP system. Based on the small group of patients that actually received BCPAP treatment, the facial mask seems feasible and effective for the treatment of acute cardiogenic pulmonary edema in the prehospital setting. These findings require confirmation in a larger prospective study.

## Abbreviations

CPAP: Continuous positive airway pressure; ICU: Intensive care unit; CCU: Coronary care unit; BCPAP: Boussignac continuous positive airway pressure; ACPE: Acute cardiogenic pulmonary edema; EMS: Emergency medical service; ED: Emergency department; CHF: Congestive heart failure; COPD: Chronic obstructive pulmonary disease.

## Competing interests

The authors declare that they have no competing interests.

## Authors’ contributions

ES collected, managed and analysed data and wrote the article. MBO conceived of and designed the study. MBA conceived of the study and was responsible for revision. MS conceived of the study, participated in its design and coordination, and was responsible for revision. KK was responsible for revision. RW provided statistical advice. KK and RW are aware they are not noted as authors. All authors have read and approved the paper. Written confirmation can be provided when necessary.
